# Supplementary material for: A Global Screen for Assembly State Changes of the Mitotic Proteome by SEC-SWATH-MS
Source: Cell Syst. 2020 Feb 26;10(2):133–155.e6. doi: 10.1016/j.cels.2020.01.001 (PMC7042714; doi:10.1016/j.cels.2020.01.001)

Q86XL3 | ANKL2_HUMAN | ANKLE2 KIAA0692 LEM4
Monomer MW [kDa]: 104.114 Monomer expected elution fraction: 40

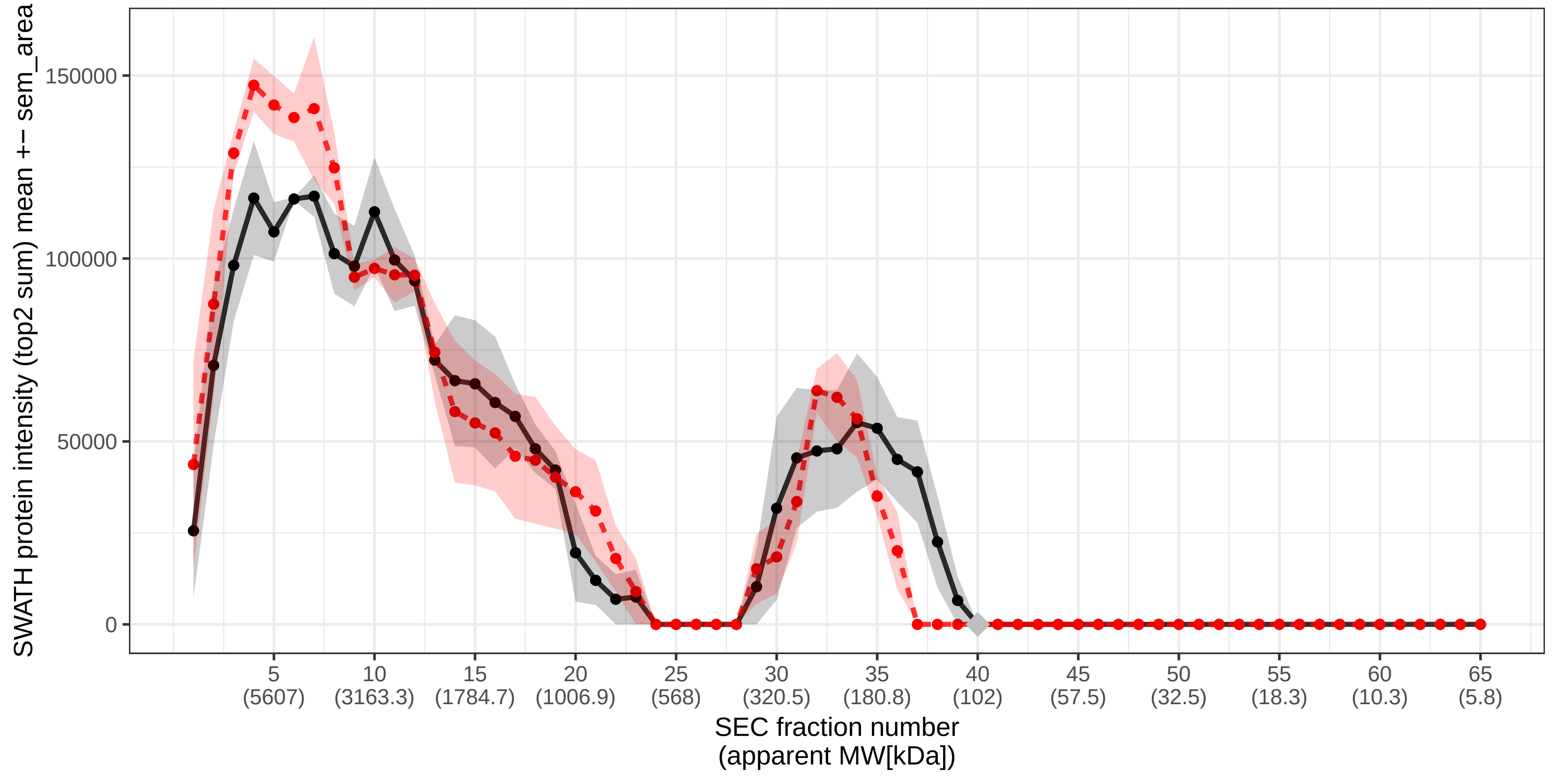

Supplement: Data S1. SEC-SWATH-MS Protein Chromatograms, Related to Figure 1 [file mmc6.zip › SECchrom_Q86XL3_ANKL2_HUMAN_ANKLE2_KIAA0692_LEM4.pdf]
